# Endotracheal Metastasis Causing Airway Obstruction

**DOI:** 10.5811/cpcem.2019.10.44964

**Published:** 2019-01-21

**Authors:** Yudai Yano, Takashi Fujiwara, Masanobu Mizuta

**Affiliations:** * Kurashiki Central Hospital, Department of Emergency and Critical Care Center, Okayama, Japan; † Kurashiki Central Hospital, Department of Otolaryngology Head and Neck Surgery, Okayama, Japan

## Abstract

Endotracheal metastasis, a critical complication of primary lung cancer, is an extremely rare lesion. A 73-year-old woman who had previously received treatment for lung cancer presented to our emergency department with dyspnea. A chest computed tomography and nasopharyngolaryngoscopy showed an endotracheal mass below the epiglottis, obstructing the trachea almost completely. The patient had an emergency tracheostomy, and then the mass was removed via median laryngotomy. This lesion was proven to be a recurrent metastasis of lung cancer. Clinicians should recognize endotracheal metastasis as an important differential diagnosis in cancer patients presenting with respiratory symptoms.

## CASE PRESENTATION

A 73-year-old woman presented to our emergency department with complaints of dyspnea for one month. She had a history of pulmonary large-cell neuroendocrine carcinoma (LCNEC) and chronic obstructive pulmonary disease. Following surgery for LCNEC, she had also completed chemotherapy and radiation therapy 10 months earlier. Currently she was receiving home oxygen therapy with nasal cannula at two liters per minute.

**Image 1:**
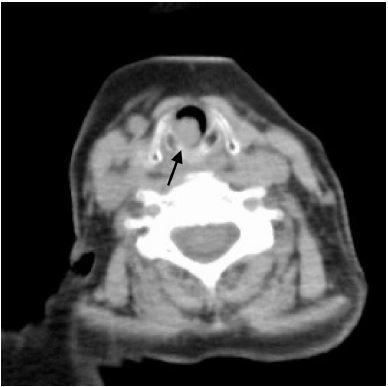
A chest computed tomography showing the endotracheal mass (arrow) immediately below the glottis.

**Image 2:**
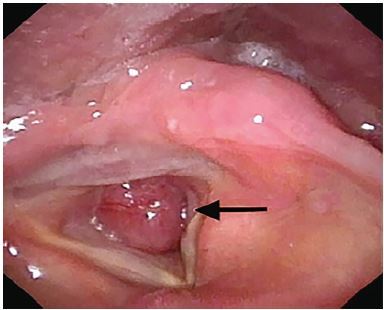
Nasopharyngolaryngoscopy showing the mass (arrow) immediately below the glottis, obstructing the patient’s airway almost completely.

Her respiratory rate was 19 breaths per minute and oxygen saturation was 96% with nasal cannula at two liters per minute. Lungs were clear to auscultation and there was no stridor. A chest computed tomography (CT) and nasopharyngolaryngoscopy showed an endotracheal mass below the glottis, almost completely obstructing the trachea (Images 1 and 2).

**Image 3:**
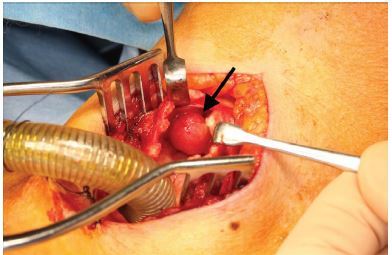
The endotracheal mass (arrow) was removed via median laryngotomy after tracheostomy.

She underwent emergency tracheostomy for airway protection, and then the mass was removed via median laryngotomy (Image 3). Post surgically, her dyspnea improved. On histopathology examination, recurrent metastasis of LCNEC was diagnosed. She was started on chemotherapy and radiation therapy for recurrent metastasis. The postoperative course was uneventful, and the tracheo-cutaneous fistula could be closed two months later.

## DISCUSSION

Endotracheal metastasis of primary lung cancer is extremely rare with a reported prevalence of 0.44%.^1^ It is a serious complication due to the risk of airway obstruction. Sudden death due to endotracheal metastasis has been reported previously.^2^ A differential diagnosis of airway metastasis should be considered in cancer patients complaining of respiratory symptoms. To avoid missing lesions of the upper airway, a chest CT including the glottis is recommended.

Depending on the patient’s general condition and site of metastasis, various treatment modalities such as airway stents, bronchoscopic extraction, laser ablation, and radiation therapy are available.^3^ However, the most important management step is to secure the airway. In our case, tracheostomy was performed to safely secure the airway because the endotracheal metastasis was immediately below the glottis. Clinicians should select the best airway management including surgical options.

**CPC-EM Capsule**

What do we already know about this clinical entity?

Primary lung cancer can metastasize in the trachea, but this is extremely rare.

What is the major impact of the image(s)?

Endotracheal metastasis of primary lung cancer obstructed the patient’s airway almost completely. After emergency tracheostomy, the metastasis was removed via median laryngotomy.

How might this improve emergency medicine practice?

Endotracheal metastasis can led to airway obstruction. Clinicians should recognize endotracheal metastasis as a differential diagnosis in cancer patients presenting with respiratory symptoms.
